# Editorial: The Dynamic Role of Suppressor of Cytokine Signaling Proteins in the Regulation of Immune and Autoimmune Responses

**DOI:** 10.3389/fimmu.2017.00825

**Published:** 2017-07-17

**Authors:** Howard M. Johnson, Joseph Larkin

**Affiliations:** ^1^University of Florida, Gainesville, FL, United States

**Keywords:** cytokine, immune tolerance, suppressor of cytokine signaling proteins, inflammation, macrophages, T cell biology

**Editorial on the Research Topic**

**The Dynamic Role of Suppressor of Cytokine Signaling Proteins in the Regulation of Immune and Autoimmune Responses**

The immune system is under both positive and negative regulation, which when functioning cooperatively, results in a state described as immune homeostasis. Positive regulation seems to be the default position of the immune system when considered in the context of several well-defined negative immune regulatory systems and families. The most familiar negative regulatory system consists of regulatory T cells as well as other regulatory lymphocytic and non-lymphocytic players (reviewed in Larkin et al.). A family of intracellular proteins called suppressor of cytokine signaling or SOCS comprises the other major regulatory system. Several genetic studies have identified variations in SOCS-related genes as risk factors in human diseases ranging from obesity to multiple sclerosis to increased susceptibility to *M. tuberculosis* infection (reviewed in McCormick and Heller). The ligand/receptor negative regulatory system known, respectively, as programmed cell death protein 1 (PD-1) and cytotoxic T cell lymphocyte antigen 1 may function within the context of regulatory cells and SOCS. The SOCS system is the major focus of the papers presented here. There are eight members of the SOCS family, described predictively as SOCS1 through SOCS7 with the eighth member called SH2 cytokine inducible protein. These different SOCS have both overlapping and non-overlapping functions with SOCS1 and SOCS3 possessing particularly importance in direct and indirect regulation of the immune system.

The role of SOCS1 in the non-redundant interdependency of the immune regulatory systems is addressed in one of the reviews of this SOCS collection along with approaches to positively and negatively regulate the activities of SOCS1 and SOCS3 (Ahmed et al.). Knockout of the SOCS1 gene is neonatally lethal in mice that uniformly die within 3 weeks of birth. Such mice are also deficient in peripheral Foxp3-positive constitutive regulatory T cells. The reviews show that a SOCS1 mimetic peptide consisting of the kinase inhibitor region (KIR) of SOCS1 in conjunction with adoptively transferred wild-type CD4 positive T cells improves SOCS1 knockout mice survival and partially restores peripheral Foxp3 T regulatory cell function. Knockdown of SOCS1 by silencing of SOCS1 expression results in inhibition of PD-1 upregulation. CTLA-4 function has similarly has shown to depend on SOCS1 in this review. The flip side to enhancement of SOCS1 function by way of SOCS1 mimetics is a review also of SOCS1 small molecule antagonist peptides that are derived from the activation loop of the tyrosine kinase JAK2, which is the binding site of SOCS1 KIR (Ahmed et al.).

An original article on the use of the SOCS1 antagonist peptide for prevention of lethal influenza A virus infection in mice is also included in the SOCS collection (Ahmed et al.). In addition to the innate immune function of the SOCS antagonist, it also possessed potent adjuvant activity against the M2e influenza virus universal vaccine antigen. Although not directly addressed in this article, the influenza virus results have potential implications for a SOCS1 antagonist approach for immunotherapy of cancer.

The role of SOCS, particularly SOCS1 and SOCS3, in regulation of macrophage, dendritic cell, and neuronal microglial cell phenotypes is comprehensively reviewed in the third contribution (McCormick and Heller). Activation phenotypes of macrophages are broadly grouped as M1 and M2 with gamma interferon (IFNγ) and toll-like receptor signaling driving M1 polarization and interleukin-4/interleukin-13 (IL-4/IL-13) functioning as the central players in M2 polarization. Functionally, M1 macrophages mediate host defense against bacterial and viral infections as well as tumors, while M2 macrophages mediate host defense against parasites and facilitate wound healing. The effect of SOCS1 and SOCS3 on M1 and M2 polarization is variable, dependent in some cases on the species of the macrophage. For example, SOC3 appears to be a positive regulator of M1 polarization in rats whereas SOCS1 may regulate both M1 and M2 polarization in rat macrophages. Mouse macrophages, in contrast, respond in an opposite manner to the SOCS molecules. In general, the SOCS tend to dampen macrophage activation. Notably, it is been recently shown that SOCS1 regulates IL-4 mediated inulin receptor substrate-2 phosphorylation in monocytes by the proteasome (1), further clarifying the role of SOCS proteins in this signaling pathway. Dendritic cells and brain microglia cells are negatively regulated by SOCS1. The microglia affect may have significance for the positive effects of SOCS1 and SOCS1 mimetics in controlling autoimmune conditions such as experimental allergic encephalomyelitis in the mouse model of the multiple sclerosis.

SOCS proteins, particularly SOCS1 and SOCS3, play key roles in pancreatic β-cell function under both physiological and pathological conditions. This is addressed in some detail in another contribution to our SOCS theme (Ye and Driver). The interactions are complex with both positive and negative effects. Case in point; SOCS1 inhibits insulin function and contributes to insulin resistance, while at the same time it is protective against autoimmune destruction of pancreatic β cells by inhibiting the activity of cytokines like IFNγ and tumor necrosis factor α (TNFα). These and other complex interactions of hormones, growth factors, cytokines, and SOCS are covered in a clear matter in the review so as to inform and suggest areas for further understanding of the role of SOCS in critical hormonal functions of pancreatic β cells and insulin.

Finally, there are two papers presented, one with original data (Atretkhany et al.) and the other a review (Bhaumik and Basu) that do not directly address SOCS function. The original paper deals with the role of TNF in the induction of myeloid-derived suppressor cells (MDSCs), and how this induction plays a role in enhanced tumor growth in mice (Atretkhany et al.). Ablation of TNF with specific antibodies reduced MDSC activity, resulting in enhanced antitumor immunity. This manuscript reminds us of the intricate interplay between cellular and molecular regulators of immune response. The review deals with the cellular and molecular aspects of T helper 17 (Th17) differentiation as well as developmental aspects of the plasticity of the Th17 in the context of intestinal immune response (Bhaumik and Basu). Although not addressed directly, SOCS proteins with their negative effects on tyrosine kinases involved in Th17 function probably play a role in this plasticity.

## Author Contributions

HJ and JL co-wrote this editorial.

## Conflict of Interest Statement

HJ and JL declare a patent pending for the use of SOCS mimetic peptides for the treatment of disease.
